# Marine algal species and their distribution in Phu Quoc marine protected area

**DOI:** 10.1016/j.dib.2019.104200

**Published:** 2019-06-28

**Authors:** Huynh Van Tien, Nguyen Tan Phong

**Affiliations:** a Faculty of Natural Resources & Environment, Kien Giang University, Viet Nam; b Faculty of Environment and Labour Safety, Ton Duc Thang University, Ho Chi Minh City, Viet Nam

**Keywords:** Marine algae, Phu quoc, Marine protected areas

## Abstract

This article presents the raw data in relation to the status of, and the distribution of, the 41 marine algal species occurring around and within the An Thoi coral reef strictly protected zone, Phu Quoc Marine Protected Area. The data, which were collected in May 2017, include the detailed description of the locations, the oceanographical conditions, and the photographs of the 41 marine algal species. For more insight, please see “Marine algal species and marine protected area management: A case study in Phu Quoc, Kien Giang, Vietnam” Huynh and Nguyen, 2019.

**Table:** Specifications table

Subject area	Biology
More specific subject area	Natural resource management
Type of data	Map, table and photographs
How data was acquired	Field visits, lab based analysis
Data format	Raw
Experimental factors	Sampling was undertaken on site
Experimental features	Algal species were sampled and stored using lab equipment.
Data source location	Phu Quoc, Kien Giang, Vietnam
Data accessibility	Data is embedded in this article
Related research article	Huynh V.T. & Nguyen, T.P. 2019. Marine algal species and marine protected area management: A case study in Phu Quoc, Kien Giang, Vietnam. *Ocean & Coastal Management***(179)**: 104816.

**Table:** 

**Value of the data** • The raw data contributes to an adequate understanding of the status of, the distribution of, 41 algal marine species occurring around and within the An Thoi coral reef strictly protected area, Phu Quoc Marine Protected Area. • The raw data forms a baseline for future zoning and zoning permits, monitoring and management of marine resources of the Phu Quoc Marine Protected Area. • The data obtained from this study permits other researchers to undertake extended analyses in relation to morphology and classification.

## 1 Data

**Fig. 1:**
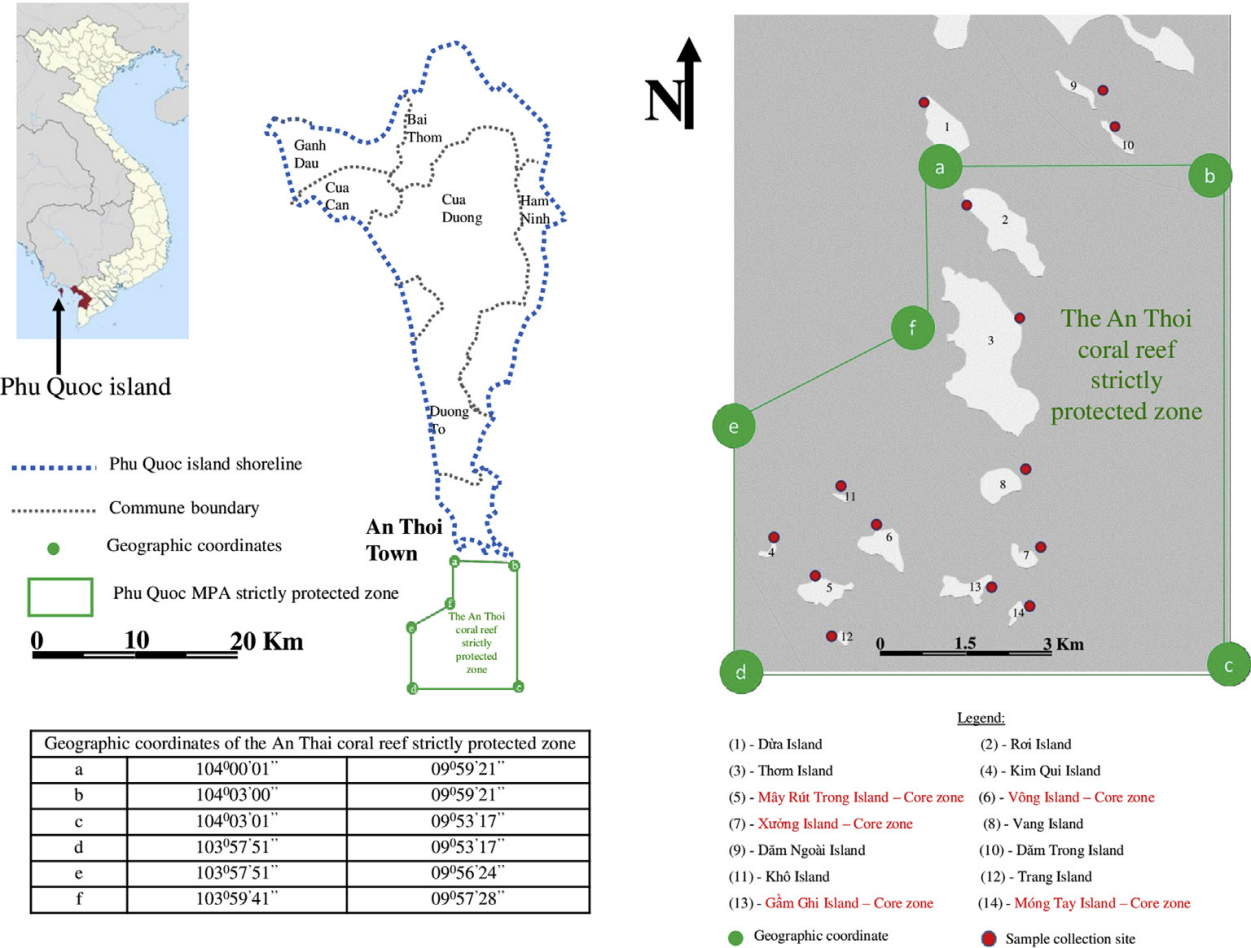
The An Thoi coral reef strictly protected area, locations of sampling [1].

**Fig. 2:**
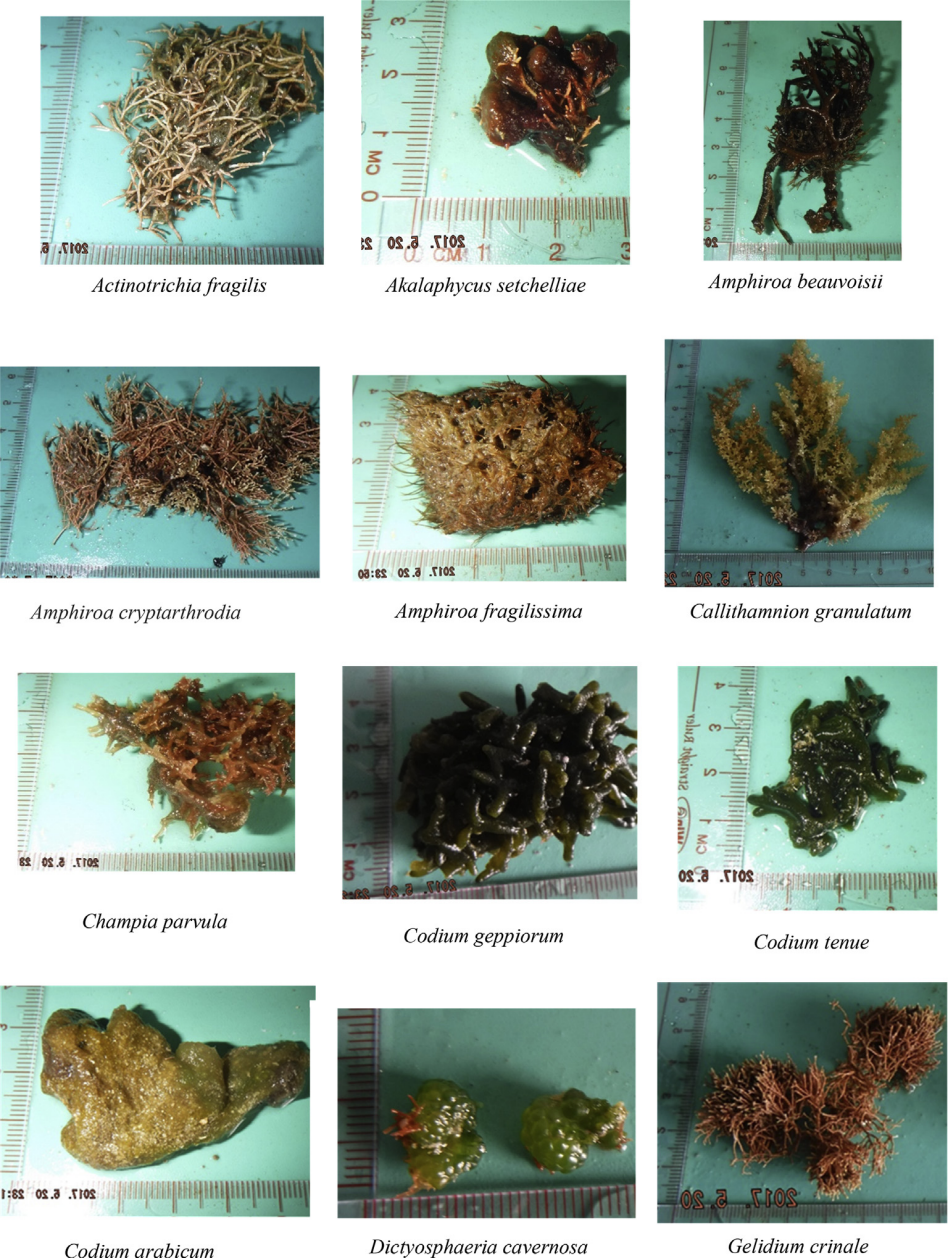

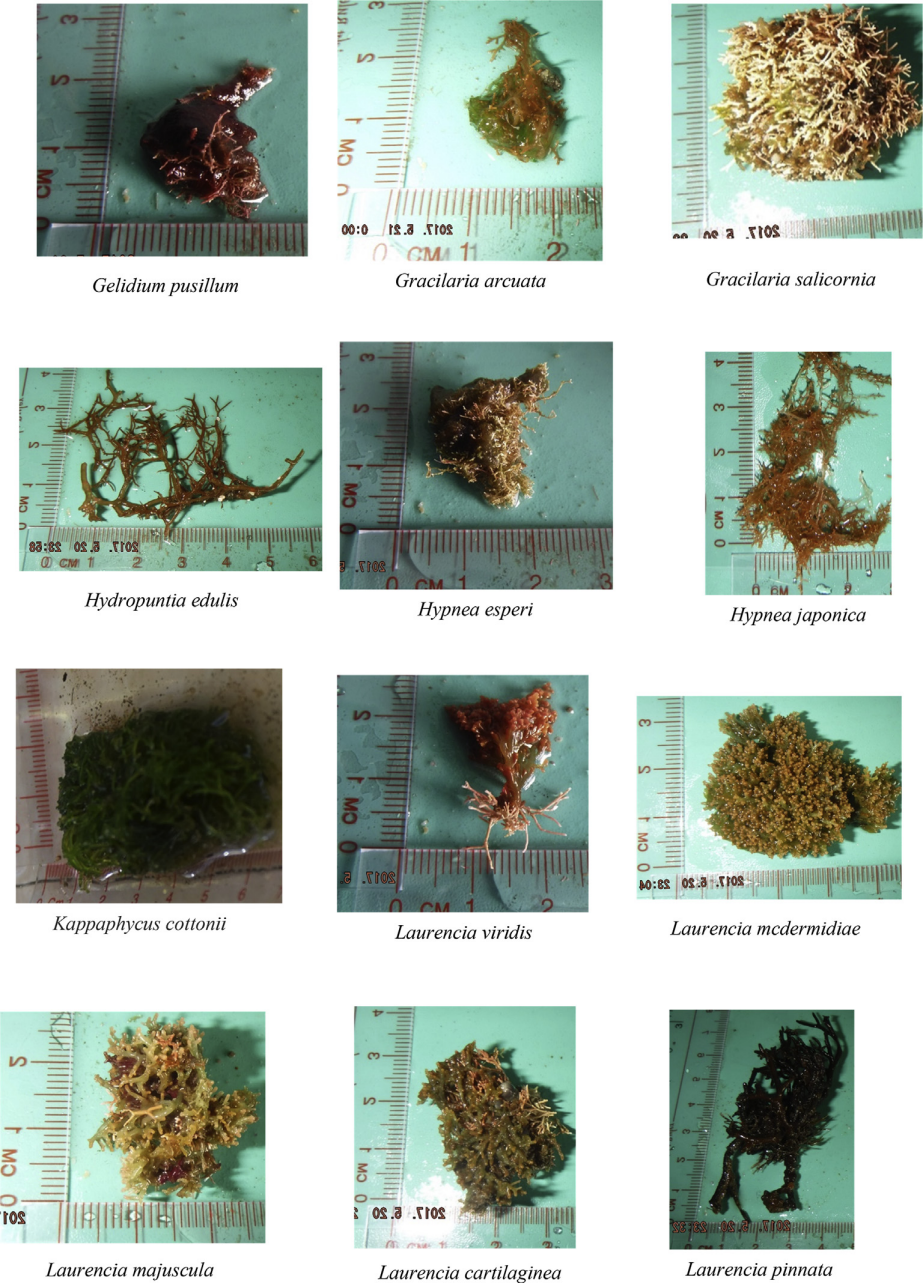

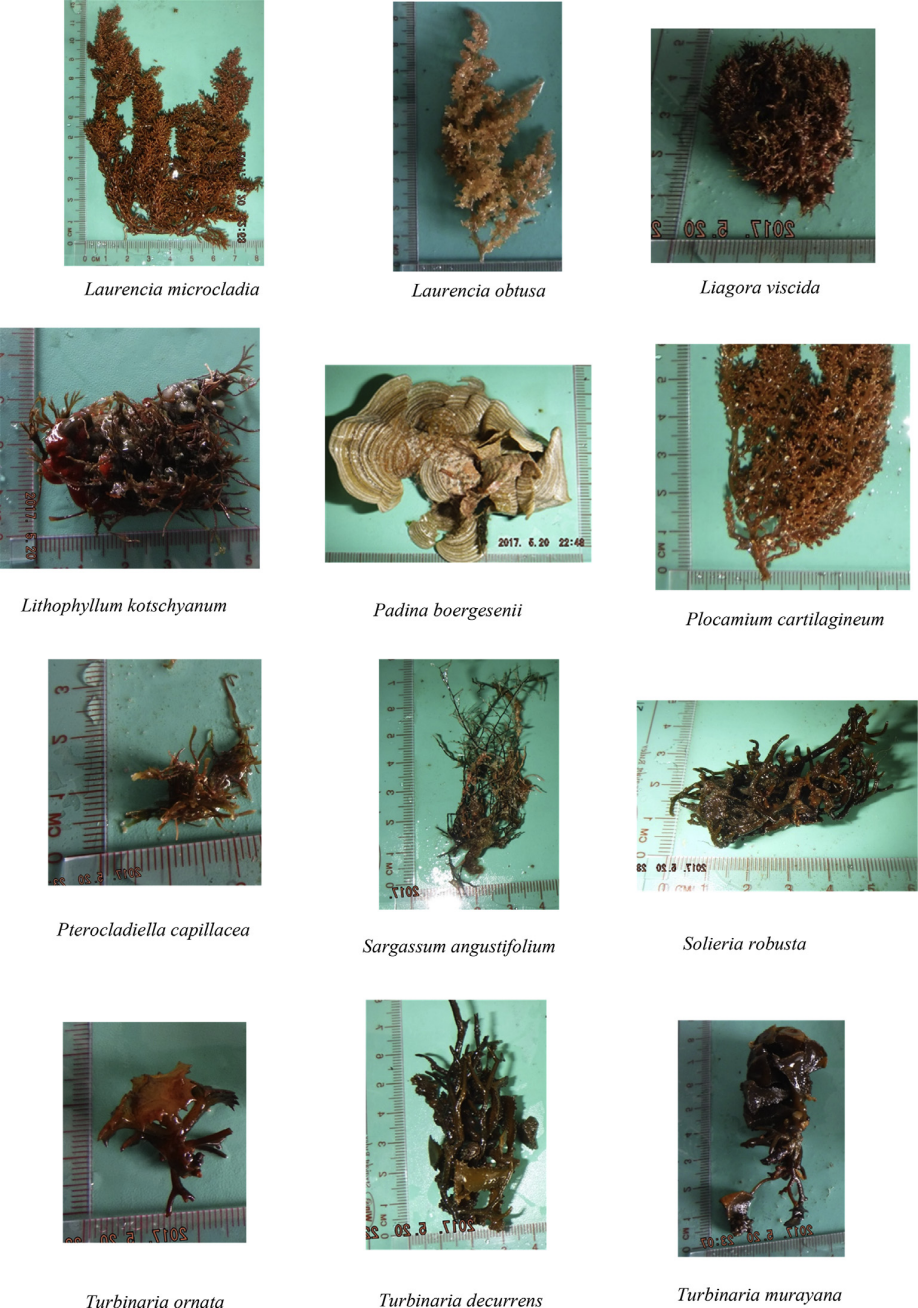

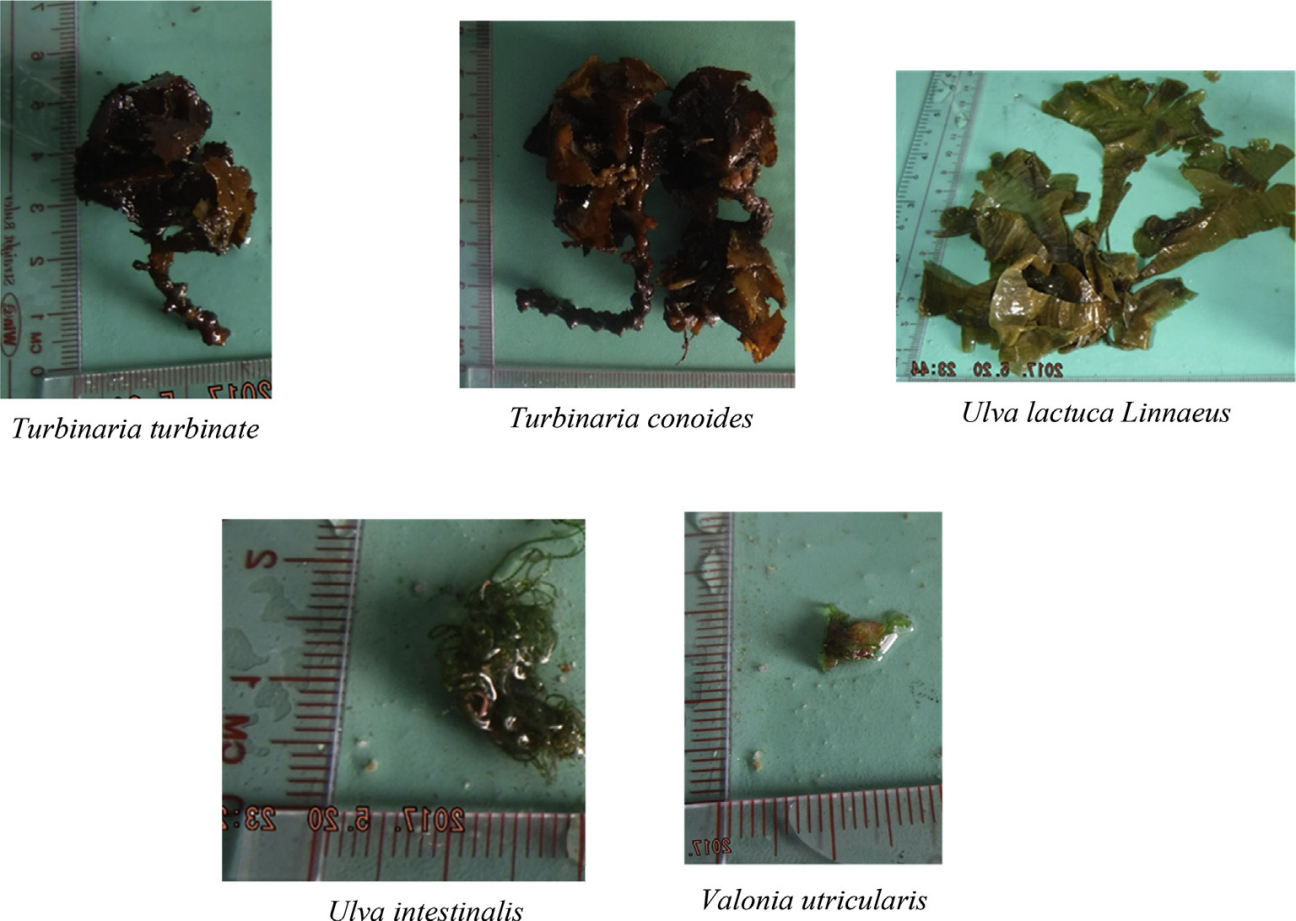
41 species recorded in the study.

The raw data include (a) the zoning map of An Thoi Coral Reef strictly protected zone, along with geographic coordinates and locations of islands around and within the protected zone (Fig. 1), (b) a table describing oceanographical conditions and species recorded at each island (Table 1), and (c) photographs of 41 algal species recorded in the strictly protected area (see Fig. 2).

**Table 1:** Physical conditions and species recorded from each island. Refer to the Fig. 1 for understanding the location numbers.

Location	Geographic coordinates	Survey date	Water temperature (°C)	pH	Salinity (%)	Depth	Species
1	9°59′36.2″N 104°00′51.6″E	05/2019	23.59	8.15	7.61	<1 m of the water surface	*Laurencia cartilaginea, Laurencia mcdermidiae, Turbinaria conoides, Turbinaria decurrens*
2	9°59′04.2″N 104°01′06.4″E	05/2019	24.61	7.92	7.82	<1 m of the water surface	*Laurencia mcdermidiae, Lithophyllum kotschyanum, Turbinaria decurrens*
3	9°57′19.4″N 104°00′46.7″E	05/2019	23.81	8.25	7.94	<1.5 m of the water surface	*Callithamnion granulatum, Hypnea esperi, Laurencia cartilaginea, Laurencia majuscule, Laurencia microcladia, Laurencia obtuse, Plocamium cartilagineum, Turbinaria ornate, Turbinaria turbinata*
4	9°55′11.1″N 103°58′52.1″E	05/2019	27.53	7.61	8.63	<1 m of the water surface	*Gracilaria Salicornia, Laurencia mcdermidiae, Lithophyllum kotschyanum, Turbinaria murayana*
5	9°54′47.2″N 103°59′40.3″E	05/2019	22.69	7.78	8.76	<0.8 m of the water surface	*Liagora viscida, Padina boergesenii, Pterocladiella capillacea, Valonia utricularis*
6	9°55′03.4″N 103°59′59.6″E	05/2019	27.94	7.91	8.57	<1 m of the water surface	*Amphiroa cryptarthrodia, Codium arabicum, Codium geppiorum, Codium tenue, Dictyosphaeria cavernosa, Gracilaria Salicornia, Laurencia pinnata, Liagora viscida, Plocamium cartilagineum, Sargassum angustifolium, Turbinaria ornate, Ulva intestinalis*
7	9°54′56.2″N 104°01′26.1″E	05/2019	27.82	8.01	8.68	<0.9 m of the water surface	*Actinotrichia fragilis, Amphiroa beauvoisii, Gracilaria arcuate, Laurencia majuscule, Laurencia mcdermidiae, Laurencia viridis*
8	9°55′38.8″N 104°01′06.7″E	05/2019	28.73	8.12	8.79	<0.9 m of the water surface	*Akalaphycus setchelliae, Gelidium crinale, Laurencia mcdermidiae, Pterocladiella capillacea*
9	9°59′28.8″N 104°02′31.3″E	05/2019	28.86	7.85	8.81	< 0.8 m of the water surface	*Champia parvula, Solieria robusta, Ulva lactuca Linnaeus*
10	10°00′20.8″N104°01′46.2″E	05/2019	24.79	7.67	8.22	<0.8 m of the water surface	*Amphiroa fragilissima, Hydropuntia edulis, Turbinaria murayana*
11	9°55′45.3″N 103°59′32.5″E	05/2019	29.63	8.08	9.03	< 1 m of the water surface	*Hypnea japonica, Laurencia obtuse, Turbinaria conoides, Turbinaria murayana*
12	9°54′13.2″N 103°59′29.8″E	05/2019	28.92	8.22	9.02	<0.6 m of the water surface	*Actinotrichia fragilis, Gelidium pusillum*
13	9°54′38.2″N 104°00′46.0″E	05/2019	28.84	7.95	8.95	<06 m of the water surface	*Gracilaria arcuate, Hydropuntia edulis, Kappaphycus cottonii, Turbinaria murayana*
14	9°54′35.2″N 104°01′20.7″E	05/2019	29.07	7.82	9.18	<0.6 m of the water surface	*Actinotrichia fragilis, Callithamnion granulatum*

## 2 Experimental design, materials and methods

**Fig. 3:**
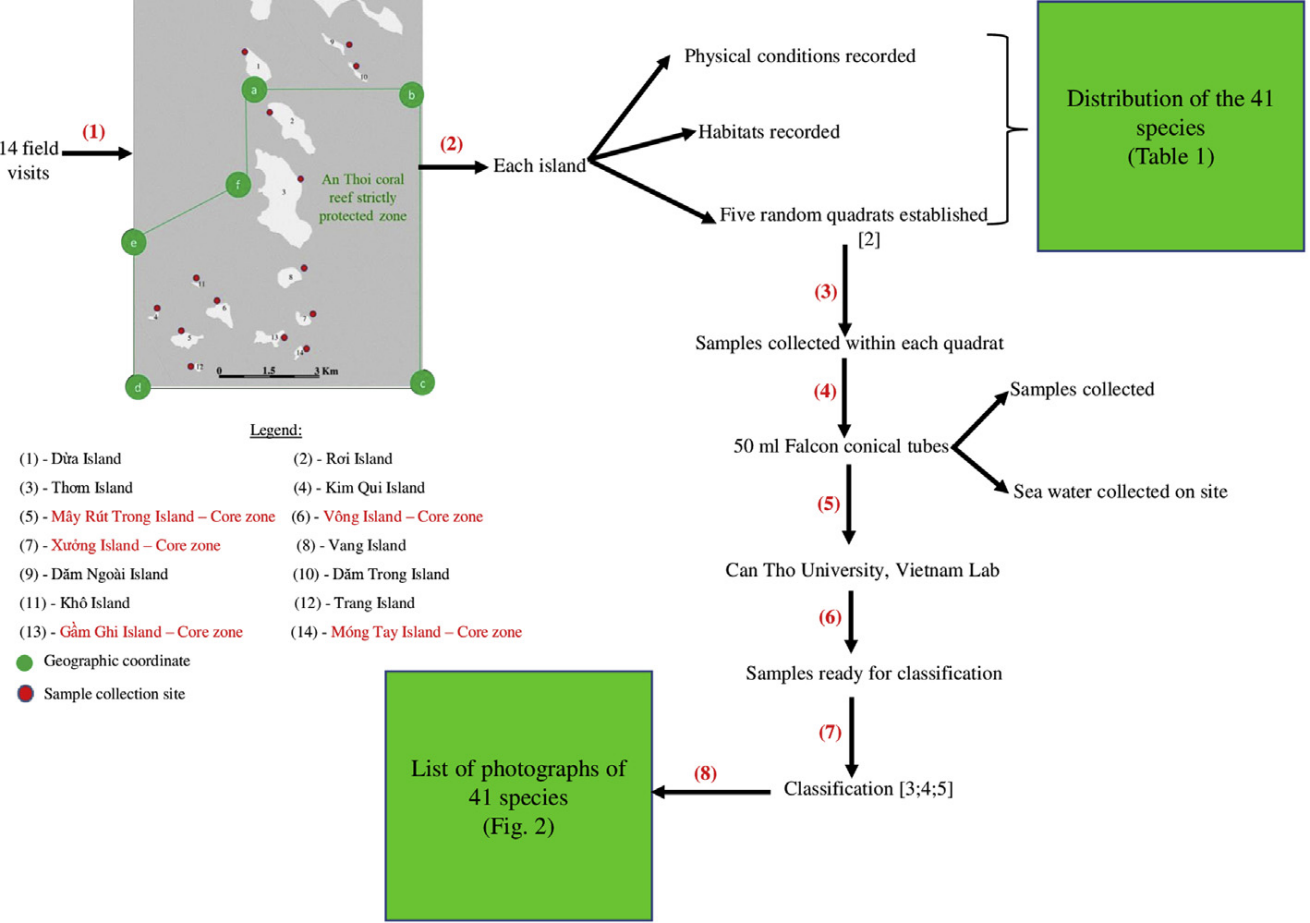
Summary of the methods used for sampling in this study.

Fourteen visits were organized in May 2017 by boat to 14 islands around and within the strictly protected area, Phu Quoc Marine Protected Area (see Fig. 1). A total of 70 samples were collected using quadrats [2]. The samples were then analyzed and identified using lab equipment in Can Tho University, Vietnam. The analysis and identification was undertaken using the recommendation suggested by [3], [4], [5]. The 8 step method is summarized in Fig. 3:
